# Viability of Open Large Language Models for Clinical Documentation in German Health Care: Real-World Model Evaluation Study

**DOI:** 10.2196/59617

**Published:** 2024-08-28

**Authors:** Felix Heilmeyer, Daniel Böhringer, Thomas Reinhard, Sebastian Arens, Lisa Lyssenko, Christian Haverkamp

**Affiliations:** 1 Institute of Digitalization in Medicine, Faculty of Medicine and Medical Center, University of Freiburg, Breisacher Straße 153, Freiburg im Breisgau, 79110, Germany, 49 27039392; 2 Eye Center, Faculty of Medicine and Medical Center, University of Freiburg, Freiburg im Breisgau, Germany

**Keywords:** machine learning, ML, artificial intelligence, AI, large language model, large language models, LLM, LLMs, natural language processing, NLP, deep learning, algorithm, algorithms, model, models, analytics, practical model, practical models, medical documentation, writing assistance, medical administration, writing assistance for physicians

## Abstract

**Background:**

The use of large language models (LLMs) as writing assistance for medical professionals is a promising approach to reduce the time required for documentation, but there may be practical, ethical, and legal challenges in many jurisdictions complicating the use of the most powerful commercial LLM solutions.

**Objective:**

In this study, we assessed the feasibility of using nonproprietary LLMs of the GPT variety as writing assistance for medical professionals in an on-premise setting with restricted compute resources, generating German medical text.

**Methods:**

We trained four 7-billion–parameter models with 3 different architectures for our task and evaluated their performance using a powerful commercial LLM, namely Anthropic’s Claude-v2, as a rater. Based on this, we selected the best-performing model and evaluated its practical usability with 2 independent human raters on real-world data.

**Results:**

In the automated evaluation with Claude-v2, BLOOM-CLP-German, a model trained from scratch on the German text, achieved the best results. In the manual evaluation by human experts, 95 (93.1%) of the 102 reports generated by that model were evaluated as usable as is or with only minor changes by both human raters.

**Conclusions:**

The results show that even with restricted compute resources, it is possible to generate medical texts that are suitable for documentation in routine clinical practice. However, the target language should be considered in the model selection when processing non-English text.

## Introduction

### Background

Physicians are often overloaded with documentation requirements, including writing a doctor’s note and a summary of a patient’s visit. An analysis of clinical software log files showed that interaction with electronic health records (EHRs) constitutes a large portion of physicians’ daily work, approximately one-fourth of which is spent writing documentation [1]. Completion of the documentation in the EHR is perceived as a tedious task, which is often done after work hours [1]. More time spent on documentation after work hours has been shown to be associated with burnout and decreased work-life satisfaction [2].

A promising approach to reduce the time required for documentation is the use of writing assistance based on large language models (LLMs). In a feasibility study, the authors trained previous-generation LLMs (GPT-2 and GPT-Neo) to complete text in medical records [3]. They concluded that the models could be used in medical charting but still have some room for improvement. A large source of error was abrupt changes in the topic, which is common in the documentation of EHRs.

With recent advances in LLM technology and the release of ChatGPT, LLMs have seen widespread adoption in assisting professionals produce text for communication or documentation purposes. For example, under the Copilot brand, Microsoft is building generative artificial intelligence (AI) capabilities into their widely used Office application suite to assist in business use cases. This leads us to believe that current-generation LLMs could also provide valuable assistance in the health care sector.

### Challenges in the Use of LLMs in the Health Care Sector

Among the best-performing LLMs, according to the continuously updated Holistic Evaluation of Language Models [4] at Stanford University, are currently commercial offerings from companies such as OpenAI or Anthropic. With these offerings, the models run on the providers’ infrastructure and are accessible via an application programming interface. However, these services cannot be used in a clinical context without further consideration.

First, in many countries, the services do not meet the legal requirements for processing protected health information. In some jurisdictions, legal and regulatory frameworks mandate that data originating from health care providers must be processed within the country’s borders or even on-premise. This is particularly problematic for European countries, as the European Union’s General Data Protection Regulation prohibits the transfer of protected health information to data centers in the United States, where most providers are located.

Second, clinical software must be thoroughly validated before it is released to end users, and in some cases, it is even subject to the Medical Device Regulation. This conflicts with the update policy of providers of commercial AI solutions. The scope of model updates is usually communicated only a few weeks in advance, for example, 2 weeks in the case of OpenAI [5]. This would not be a problem if these updates were only additive in functionality, but the opaque nature of current LLMs also means that improvements to some aspects of model performance might unexpectedly negatively affect the performance on other tasks [6]. The use of fixed model versions, as offered by some providers, is not practicable in the long term, as older models are often removed after the release of updates; in the case of OpenAI, after 3 months [5].

### Training Nonproprietary AI Models for Medical Text

An alternative is the use of nonproprietary AI models. In these models, the architecture as well as the trained parameters are available to the user. This solves the aforementioned problems by giving the user the option to train and deploy these models on any infrastructure and fully control any changes to it.

One of the largest pretrained LLMs is GPT models that enable model training with limited data sets. There are several approaches to applying GPT models to a task. One common approach is to use a very large model that is trained primarily with general text corpora and includes instructions for the task in the input for the model, the so-called prompt. This is sometimes called in-context learning (ICL) or, depending on whether examples are provided, zero-shot or few-shot learning.

ICL works reasonably well on tasks that have a good representation in the base models’ training corpus. However, the structure and content of clinical notes differ significantly from the general-purpose text corpora used to train most publicly available LLMs. Even including biomedical text from publications, such as PubMed papers, in the training data could only have minor effects on model performance compared to training on clinical text [7-9]. Lehman et al [9] compared ICL and multiple alternatives such as (1) training from scratch on a clinical corpus, (2) continuing training a pretrained model on the clinical text and then fine-tuning for the downstream task, or (3) directly training the GPT for the downstream task without further pretraining. They show that relatively small specialized clinical models substantially outperform all ICL approaches and conclude that pretraining on clinical text allows for smaller, more parameter-efficient models.

One fact that must be taken into account when using GPT models in a clinical context is that the pretrained models have now become very large. Complete fine-tuning, in which all model parameters are retrained on the task-specific data, is therefore becoming less and less feasible. This is particularly the case if the models have to be trained on site for legal or economic reasons. The computing power available here is usually limited, which restricts the size of the models that can be trained. Accordingly, the choice of models is a trade-off between training time and costs, model accuracy, and maximum sequence length.

One possibility to address the problem of limited working memory is the Low-Rank Adaptation (LoRA) technique [10]. Here, all the model weights are frozen, and only a few very small additional low-rank matrices are added to the query and key parameter matrices of the transformer attention heads and subsequently optimized. This reduces the number of trainable parameters by 10,000 times and the graphics processing unit (GPU) memory requirement by 3 times. Recently, training of quantized models became possible by combining LoRA with quantization [11]. With Quantized Low-Rank Adaptation (QLoRA), a frozen quantized model is fine-tuned by optimizing added low-rank adapters at 16-bit floating-point precision. The QLoRA technique also introduced additional memory-saving mechanisms such as the 4-bit Normal Float (NF4) data type for quantization and paged optimizers [11].

### Aim of This Study

In this study, we assessed the feasibility of using nonproprietary LLMs of the GPT variety as writing assistance for medical professionals in an on-premise setting with restricted compute resources, generating non-English medical text. We trained 4 models with 3 different architectures for our task using the Hugging Face Transformers framework [12] and explored their performance using a powerful commercial LLM, namely Anthropic’s Claude-v2, as a rater. Based on this, we selected the best-performing model and evaluated its practical usability with 2 independent human raters on real-world data.

## Methods

### Ethical Considerations

The study was implemented in the outpatient clinic of the Eye Center at Medical Center, University of Freiburg, Germany, and was approved by the responsible ethical review committee (registration 23‐1444S1). All data used in the study were deidentified and contained no references to patients and practitioners. Informed consent for the anonymization process was not obtained. Data processing was justified based on the legal basis of “legitimate interest” in accordance with the General Data Protection Regulation and the state hospital law of Baden-Württemberg (“Landeskrankenhausgesetz”). Participants did not receive any financial compensation for their data use, as the data were retrospectively reviewed from existing medical records.

### Study Design

The target for assistive text generation was the final part of the medical documentation of an examination or treatment, the so-called epicrisis report. In this report, the doctors write a structured compilation of the information so far documented in the EHR in text form. It contains the relevant medical information of the case and usually consists of three sections: (1) main diagnosis or the patient’s reason for visit, (2) therapeutic procedures or medication, and (3) recommendations for further intervention and need for a follow-up appointment.

### Data

#### Data Source and Description

The data pool used for training the models was the EHR records of 82,482 unique patient encounters that span approximately 10 years of clinical practice. The EHR record of an encounter contains all digital information about a patient’s examination or treatment in the outpatient clinic, which offers specialist, emergency, and follow-up care. The data are collected in various ways over the patient’s visit. Support staff record basic information in structured forms, doctors document the medical history, symptoms and previous or planned treatments are documented in text notes, and diagnostic data from electronic devices are mainly stored in numeric format. The final epicrisis report consists of a stand-alone text, which is filed alongside all other information in the EHR record.

The whole training data set amounts to approximately 140 MB of uncompressed text in Unicode Transformation Format 8 encoding or approximately 29 to 33 million tokens, depending on the tokenizer model used. A data set of 509 patient encounters that occurred after the training set date cutoff was set aside for comparison of model performance in the evaluation. The complete data set consists of German text. The examples used in this paper were translated into English by the authors of the paper.

#### Preprocessing and Formatting

For the LLM training, all available data in the EHR record were concatenated into 1 continuous text sequence per encounter. The types of information were separated by newlines and prefixed with a descriptor such as “History” or “Pressure Measurement” to form the prompt. If no data were documented in a section, it was left empty. The order of sections matched the order in which the fields are displayed to users in the EHR software interface. The last section of each text sequence was the epicrisis report. If there were separate records for each eye, the individual records were additionally prefixed with an abbreviation indicating the side.

Task training was implemented by inserting special tokens to mark the text to be generated by the final models, that is, the epicrisis report. Each text sequence starts with a special token indicating the beginning of the input data recorded during the patient visit, that is, all other information in the EHR record. A second special token is inserted before the epicrisis report, indicating the start of the generation task. In the training data, this token is followed by the actual report of the attending physician. The text sequence ends with a “Stop of Sequence” token, which indicates that the model should end the generation process.

For instruction-tuned models, the text sequence was prefixed with a so-called system message enclosed in special tokens indicating instructions for the model, reading as follows: “You are an experienced doctor in a German eye hospital. Your writing style is concise, accurate, and respectful. You are writing a short note in German to a colleague about a patient. The letter should contain the provided information.”

### Models

#### Model Selection Overview

In the selection of models from openly available pretrained models, we considered hardware costs, feasibility of the training process, language aspects, and performance benchmark results, such as Stanford’s Holistic Evaluation of Language Models [4] and the Open LLM Leaderboard on Hugging Face [13]. Most LLMs are predominantly trained in English texts, and currently, there is no model that contains a greater amount of medical text. Consequently, we chose the following 3 models: LLaMA, LLaMA-2-Chat, and BLOOM-CLP-German.

#### LLaMA

At the start of this study, Meta AI’s LLaMA model was among the top performers on several open LLM benchmarks. In contrast to some of its competitors, its training corpus also contains some German text but no clinical content [14]. Since then, more powerful models have been released, but LLaMA still achieves competitive results on many benchmarks.

#### LLaMA-2-Chat

During our experiments, Meta AI released the successor to LLaMA [15]. Together with the updated base model, they also released an instruction-tuned model aligned with human preferences using reinforcement learning, similar to how ChatGPT was based on GPT-3 [16]. We chose this model to investigate the potential advantage of using an instruction-tuned model.

#### BLOOM-CLP-German

This model is designed for tasks in German based on the BLOOM architecture from BigScience Workshop et al [17]. It was initialized with the novel cross-lingual and progressive transfer learning (CLP) technique [18], which uses information from a small model trained in a target language and a larger model in a source language. This considerably reduces the training needed to achieve performance on par with that of a model trained from scratch. Although the model is still severely undertrained for its size [19], we included it to study the potential performance gains achieved by a model with a training corpus closer to the target text material.

### Training

#### Overview

We restrict our training setup to 8x NVIDIA RTX 3090 24-GiB consumer-grade GPUs in a single host. We load and train our models using the “transformers” Python library by Hugging Face [12] with the PyTorch [20] backend. Data are preprocessed using Hugging Face’s “datasets” Python library [21]. Distributed training on multiple GPUs is implemented via the “accelerate” Python library [22].

For each training process, we randomly sample 5% of the training data as validation data. We regularly evaluate training loss on the validation set during training, about 20 times per epoch. We stop training when the validation loss does not improve in 10 evaluation steps. This amounts to around 13 epochs for most models.

#### Memory Optimization

For fine-tuning the model for our task, we use the LoRA at full 16-bit precision and QLoRA [11] at reduced NF4 precision techniques. Reducing the precision also reduces the memory use and allows for longer input text sequences with the available memory. With this, we explore the trade-off between computational precision and input context size.

Additionally, we use 2 methods to trade reduced memory requirements for computation time. First, we use gradient checkpointing, a technique that recomputes some network activations during the backward pass on the fly instead of caching them in memory. Second, we use the Zero Redundancy Optimizer technique [23], which includes memory savings achieved by reducing redundancy when training on multiple GPUs as well as offloading some tasks to the CPU, both at the cost of communication overhead. Both make the training process considerably slower but should not impact the task performance of the resulting model.

Specifically, we trained the following model variants: LLaMA with LoRA at floating point 16-bit precision, LLaMA 2 Chat with QLoRA at NF4 precision, BLOOM-CLP German with QLoRA at NF4 precision, and BLOOM-CLP German with LoRA at floating point 16-bit precision.

### Inference

#### Overview

At their core, the decoder part of the transformer architecture models a probability distribution for the next token, given a sequence of input tokens. Both the composition of the initial input tokens and the method of choosing the next token from the produced probability distribution can have a big impact on the quality of the final result.

#### Completion Prefixing

At inference time, the model receives an input text sequence, often called the prompt. It consists of the input data, as described in the “Preprocessing and Formatting” section, followed by a special token, indicating that the subsequent text should be an epicrisis report. In other words, the model receives a text sequence containing all information from an EHR record except for the attending physician’s epicrisis report and is asked to write this report, that is, to generate a text that corresponds in content, structure, and form to the epicrisis reports included in the training data. However, in the qualitative analysis of our initial findings, we found that in some cases the models attempted to continue with the recorded data rather than start writing a final report.

In an effort to improve results without retraining our models, we introduce a simple form of prompt tuning by adding a static suffix to the prompt, that is, forcing the model to begin the generated text with the words “During today’s visit....” This suffix represents the typical beginning of the epicrisis report, as almost all reports written by doctors in the training data set start with some variation of these words. We hope that this gives the models an additional signal to complete the text with a summary and recommendations instead of trying to invent more “facts” about the patient’s stay. We report and compare the evaluation results on reports generated with and without the completion prefix.

#### Contrastive Search

For a given input sequence, the trained transformer model produces a probability distribution for the next token. Simply choosing the token with the highest probability often produces text that lacks coherence and diversity. Techniques that maximize the probability over multiple tokens (eg, beam search) or stochastic sampling can enhance coherence and diversity but are not targeted at the problem of repetition that is common to the type of highly standardized text generated in this study. We therefore use a more recently introduced technique, called contrastive search, which has been shown to encourage diversity and produce coherent results while reducing repetitiveness [24,25].

### Evaluation

#### Overview

Evaluating the quality of generated natural language text using (preferably multiple) human raters is costly and time-consuming, especially, if the rating process requires specialized domain knowledge as in this study. On the other hand, there is no obvious way to automate this process. An interesting idea is to use larger and more powerful language models to rate the quality of the output. This technique has recently been used in some publications in the LLM space, for example, in the creation of the LLaMA-2 model and in evaluating the performance of QLoRA training [11,15]. Large commercial language models such as OpenAI’s GPT-4 and Anthropic’s Claude-v1 model have been shown to achieve agreement rates with human raters of up to 80% when evaluating the output of other models [26].

#### Automated Evaluation With Claude-v2

We evaluate the generated text in a 2-step process using Claude-v2 by comparing the generated text to the epicrisis reports that were written by physicians for 509 individual patient encounters. In the first step, we extract the text passages that contain relevant information for each of the three main categories of information: (1) main diagnosis or patient’s reason for visit, (2) therapeutic procedures or medication, and (3) recommendations for further intervention and need for a follow-up appointment. In the second step, for each case and category separately, we ask Claude to evaluate whether the extracted passage from the generated report matches the passage extracted from the report written by a human.

#### Human Evaluation

The suitability of the generated text by the best-performing model is evaluated by 2 independent expert senior physicians. For this purpose, the raters are presented with the basic data from the documentation of 102 patients as well as both versions of the report: the one written by the attending physician and the computer-generated version. The raters assess whether the computer-generated version is suitable as a text template and could be used without major changes.

## Results

### Model Performance

Table 1 shows the percentage of reports in the test set in which the models matched the extracted diagnosis, follow-up, and therapy recommendation. The highest agreement rates with reports written by a doctor were achieved by the BLOOM-CLP-German model, followed by LLaMA-2 and LLaMA. The ranking was consistent across all the diagnosis, follow-up, and therapy dimensions. On average, the models achieved the highest scores in the diagnosis dimension, followed by the therapy and follow-up dimensions.

Of the BLOOM-CLP-German variants trained with full floating point 16-bit precision LoRA and reduced NF4 integer precision QLoRA, the latter achieved slightly higher agreement rates. In contrast to our intuition, prefixing the model prompt at inference time (see “Completion Prefixing” section) slightly reduced the performance across all models rather than improving it.

**Table 1.** Fraction of reports in the test set where the models match the information extracted from the text written by a doctor.

Category	Model (%)								
	BLOOM-CLP-FP16	BLOOM-CLP-FP16-prefix	BLOOM-CLP-QLoRA	BLOOM-CLP-QLoRA-prefix	LLaMA-2-QLoRA	LLaMA-2-QLoRA-prefix	LLaMA-FP16	LLaMA-FP16-prefix	Mean (SD)
Diagnosis	50.10	44.01	55.40	45.78	34.38	32.81	31.24	16.50	38.78 (12.47)
Follow-up	41.45	32.02	43.42	33.79	36.94	31.24	34.97	21.02	34.36 (6.90)
Therapy	43.81	37.33	50.69	42.44	36.54	36.35	29.67	13.95	36.35 (10.99)
Mean (SD)	45.12 (4.47)	37.79 (6.01)	49.84 (6.04)	40.67 (6.01)	35.95 (1.38)	33.46 (2.62)	31.96 (2.72)	17.16 (3.58)	—^a^

a Not applicable.

### Human Evaluation

A total of 102 reports generated by the BLOOM-CLP German model trained with QLoRA at NF4 precision were rated for suitability by 2 independent expert senior physicians. Of the 102 reports, 95 (93.1%) were evaluated as suitable by both raters, which means that computer-generated reports could be used in this form or with minor changes. Only 7 (6.9%) of the reports were rated as unsuitable by at least 1 of the raters. Cohen κ was run to determine the interrater reliability. There was moderate agreement between the 2 physicians’ judgments (κ=0.582, 95% CI 0.217-0.947; *P<.*001).

The 7 reports that were rated as unsuitable show different anomalies. In 3 of the reports, the model was caught in a loop of repeating nonsensical word sequences, for example, “we recommend local therapy with Bepanthen eye ointment 5x daily on both sides for 5‐7 days, then 1x daily on both sides for 5‐7 days, then 1x daily on both sides for 5‐7 days, then 1x daily on both sides for 5‐7 days, etc” (26 repetitions). In one case, there is no text output because the patient’s appointment did not take place. Only in 3 reports are content-related aspects decisive. In one case, the main diagnosis is not mentioned; in one case, information is missing in the treatment recommendation; and in one case, the time given for the follow-up appointment is incorrect.

## Discussion

### Principal Findings

Despite being severely undertrained compared to both LLaMA models, the BLOOM-CLP-German model achieved the best performance in our experiments. This suggests that a better alignment of the base model with the reports’ language might be more important than a longer training time. We speculate that the German vocabulary in the model’s tokenizer better-captured domain semantics compared to the multilingual tokenizers. Additionally, the model might have profited from a larger maximum input sequence length, given the limited memory. This is an effect of the smaller token per character ratio of a tokenizer with a better alignment to the text’s language.

Because its vocabulary is closer to our data, BLOOM-CLP-German’s tokenizer encodes up to 30% fewer tokens for the same input text compared to LLaMA’s tokenizer. This means that we can fit more information into the context window, training and inference consume about half as much memory, and inference is about twice as fast. This makes for significant cost reductions compared to models with a multilanguage tokenizer.

Of both BLOOM variants trained with LoRA and reduced QLoRA precision, the latter performed better in our analysis. This suggests that the reduced precision is more than offset by the bigger maximum input sequence length, given the memory constraints. We surmise that capturing more context in the model input outweighs compute-optimal training or precision.

In contrast to our intuition, forcing the models to start the generated text with a predefined prefix did not improve the results. We speculated from our manual testing that this technique might eliminate some edge cases where the models sometimes start generating text completely unrelated to the input sequence. While this might still be true, the prefix also might have impacted the models’ ability to flexibly react to the input and therefore reduced quality in more cases than improving it.

### Feasibility of Nonproprietary On-Site AI

Our manual evaluation clearly shows that it is possible to provide helpful writing assistance using nonproprietary on-site AI technologies. Most of our test samples were rated useful as is or with only minor modifications. Additionally, qualitative analysis of samples rated as unusable showed that these were edge cases where the model produced no output or text that was easily identifiable as an anomaly. Only in very few reports were content-related aspects decisive, that is, the model omitted major details or produced factually incorrect information.

Legal and ethical concerns, as discussed in the “Challenges in the Use of LLMs in the Health Care Sector” section, currently may prevent many health care providers in European countries from using proprietary AI assistance for charting. Nonproprietary models, as used in this study, allow for flexible model deployment to comply with data protection requirements. Full control over the model also addresses legal concerns regarding software certification and some ethical concerns because these models can be more easily inspected regarding potential biases. Therefore, the approach presented in this study should be feasible for most health care providers.

In this study, we chose model sizes around 7 billion parameters. In comparison, GPT-3, the model that powered the first version of ChatGPT, has 175 billion parameters. With careful optimization of trade-offs between training time and cost, model precision, and maximum sequence length, we show that it is still possible to provide helpful writing assistance even with a much smaller model. At our chosen model scale, with around 7 billion parameters operating, the models should be economically accessible to many health care providers or local service providers, making it easier to comply with local regulations and reducing possible dependence on external or foreign service providers.

### Limitations

Due to the limited availability of compute time, we were unable to test all combinations of model and training modalities. LLaMA-2 was only trained using QLoRA, and LLaMA only using LoRA, limiting possible comparisons between the base models. Similarly, we only included the instruction-tuned variant of LLaMA-2 and cannot compare to the base model without instruction tuning.

The limited training of the BLOOM model probably affected its accuracy. However, this limitation highlights the importance of language alignment with the undertrained BLOOM model, outperforming both LLaMA models.

While our human raters evaluated our chosen model’s outputs favorably, this happened in dedicated research settings. This means that the contextual information available to the human raters was restricted to the limited information included in the study data set. It remains to be shown whether AI writing assistance is still perceived as helpful in a real clinical setting or if the additional mental load caused by having to check the AI’s output in a more complex case outweighs its usefulness.

### Future Work

Moving forward, leveraging German clinical corpora for pretraining could provide useful in-domain semantics. Techniques such as CLP fine-tuning can enable the use of such data with lower compute requirements. In a future study, we will explore the use of our models in a real-world setting.

### Conclusions

This work demonstrates the feasibility of localized AI assistance for clinical note generation using small-scale nonproprietary models. Our results highlight the advantages of language-specific model tuning, providing a promising direction for future research, especially when considering the significant speed and cost advantages of the language-specific model.

## Abbreviations

AI: artificial intelligence
CLP: cross-lingual and progressive transfer learning
EHR: electronic health record
GPU: graphics processing unit
ICL: in-context learning
LLM: large language model
LoRA: Low-Rank Adaptation
NF4: 4-bit Normal Float
QLoRA: Quantized Low-Rank Adaptation
